# Feasibility and preliminary effectiveness of a highly intensive inpatient treatment programme with Narrative Exposure Therapy for patients with posttraumatic stress disorder

**DOI:** 10.3389/fpsyg.2025.1516144

**Published:** 2025-05-29

**Authors:** Patricia J. M. Strijk, Mirjam J. Nijdam, Ellen R. Klaassens, Viyan Bedawi, Simone de la Rie, Ruud A. Jongedijk

**Affiliations:** ^1^ARQ Centrum’45, partner in ARQ National Psychotrauma Centre, Oegstgeest, Netherlands; ^2^Department of Psychiatry, Amsterdam University Medical Centers Location University of Amsterdam, Amsterdam Public Health, Amsterdam, Netherlands

**Keywords:** Narrative Exposure Therapy, massed therapy, PTSD, treatment resistance, pilot, feasibility

## Abstract

**Introduction:**

Current guidelines recommend trauma-focused therapies for the treatment of post-traumatic stress disorder (PTSD). Unfortunately, the percentage of non-response and dropout with these forms of therapy has proven to be substantial. Trauma-focused therapies offered in a highly intensive format have been found to lead to higher adherence and faster symptom reduction, but no studies so far have investigated Narrative Exposure Therapy (NET) in an intensive programme. The aim of this study was to investigate the feasibility and preliminary effects of a 10-day highly intensive inpatient treatment programme consisting of Narrative Exposure Therapy (NET), Art Therapy (AT) and trauma sensitive yoga (TSY) for patients with severe, chronic PTSD.

**Methods:**

A non-controlled pilot study was conducted in which 28 individuals diagnosed with PTSD received this highly intensive treatment programme. Outcome measures were administered at baseline, post-treatment and at four-month follow-up. Changes in clinician-rated and self-reported PTSD were explored, as well as changes in depressive symptoms and general psychopathology. Outcome measures were the Clinician-Administered PTSD Scale for DSM-5 (CAPS-5), PTSD Check List for DSM-5 (PCL-5), Beck Depression Inventory (BDI-II), and Brief Symptom Inventory (BSI).

**Results:**

From baseline to four-month follow-up, clinician-rated and self-reported PTSD symptom severity significantly decreased with a large effect size (Cohen’s *d* 0.80 and 0.84, respectively). Depressive symptom severity showed a medium-sized decrease at post-treatment, which was enhanced at four-month follow-up representing a large effect (Cohen’s *d* 0.78). The drop-out rate was 18%.

**Conclusion:**

The findings of this study show that applying NET in a highly intensive form is feasible and significant improvements in PTSD and depressive symptoms were demonstrated in patients who had all received previous treatment for their condition. Future research into this promising approach is encouraged, as well as investigating which patients benefit most from highly intensive programs with NET.

## 1 Introduction

In European countries, at least 80% of people in the general community will be exposed to one or multiple traumatic events in their life (Hoeboer et al., 2025; Einarsdottir et al., 2023; Hyland et al., 2021). In persons exposed to trauma, the risk of developing posttraumatic stress disorder (PTSD) is around 4% (Kessler et al., 2017). Individuals with PTSD experience intrusive symptoms such as flashbacks and nightmares, avoidance of trauma-related stimuli, negative thoughts and beliefs about themselves or the world, and heightened arousal and reactivity (American Psychiatric Association, 2013). PTSD is a mental health disorder leading to serious levels of impairment, high risk of suicide attempts and high costs for society (Kessler, 2000). Cumulative trauma is associated with increased PTSD severity (Robjant and Fazel, 2010). Furthermore, PTSD patients who have been exposed to multiple traumas are at greater risk of developing comorbid somatic and psychiatric disorders (Karatzias et al., 2019).

Current guidelines for PTSD recommend trauma-focused treatment (TFT) for PTSD as first line of treatment (American Psychological Association, 2017; Akwa GGZ, 2020; National Institute for Health and Care Excellence, 2018). Several evidence-based TFTs are recommended by guidelines, such as Prolonged Exposure Therapy (PE; Foa et al., 2019), Cognitive Processing Therapy (CPT; Resick et al., 2017), Cognitive Therapy (CT; Ehlers and Wild, 2020), Eye Movement Desensitization and Reprocessing (EMDR; Shapiro, 2018), Brief Eclectic Psychotherapy for PTSD (BEPP; Gersons et al., 2015), and Narrative Exposure Therapy (NET; Schauer et al., 2011). All these therapies have been proven to be effective in reducing PTSD symptoms with good effect sizes (Hoppen et al., 2024; Bisson and Olff, 2021). Despite the overall efficacy of all guideline-recommended PTSD treatment options, not all patients benefit equally. A high proportion of traumatised patient groups such as refugees (18–54%) and veterans (up to 66%) retain their PTSD diagnosis after TFT (Haagen et al., 2017; van Gelderen et al., 2020) and the majority has considerable residual symptoms (Bisson et al., 2013; Bradley et al., 2005). The limited efficacy of TFTs for considerable groups of PTSD patients and the high dropout rates calls for efforts to improve the treatment of PTSD.

Intensive or massed treatment methods, in which patients undergo TFT in a condensed form over a period of 1 to 3 weeks, appear to be a promising strategy for tackling problems such as behavioural and experiential avoidance and non-adherence (Badour et al., 2012; Held et al., 2020). Currently four randomised controlled trials have been conducted on short, intensive forms of prolonged exposure and cognitive therapy for PTSD as compared with regular weekly treatment, and have shown similar efficacy (Ehlers et al., 2014; Foa et al., 2018; Oprel et al., 2021; Dell et al., 2023). The pooled dropout rate across studies showed that 5.51% of the participants dropped out of an intensive intervention, a rate that is significantly lower than in studies of standard trauma-focused treatments (Sciarrino et al., 2020). Uncontrolled studies into the effects of highly intensive treatment with exposure and EMDR have shown large effects for patients with PTSD including those with complex forms of the disorder (Voorendonk et al., 2020; Zepeda Méndez et al., 2024), the dissociative subtype of PTSD (Zoet et al., 2018; Zepeda Méndez et al., 2024), and those who had received previous ineffective TFT (Zepeda Méndez et al., 2018). In other words, complexity does not appear to be a contraindication for these programs. Apart from a recent case series on a one-week form of NET with 6 sessions that showed promising reductions in PTSD and related symptomatology (Miller et al., 2024), no studies have systematically evaluated intensive NET.

NET has specifically been developed for the treatment of multiple traumas across the patient’s lifespan (Schauer et al., 2011) and is often applied to patients in vulnerable living circumstances. In NET, imaginal exposure to the traumatic events is employed with the aim of reorganising memories within the trauma network and embedding these memories in the autobiographical context (Schnyder et al., 2015). At the start of therapy, the patient’s lifeline is symbolically laid out by means of a rope, with several symbols such as flowers and stones placed next to this rope. The lifeline is worked through in chronological order, with a testimony report being written by the therapist for each session. Four to twelve NET sessions of 90 min are recommended, depending on the number of traumatic events the individual has been exposed to Schauer et al. (2011). Significant symptom reduction was shown to result from NET even in the presence of considerable levels of psychosocial stress (Lely et al., 2019; Siehl et al., 2021) and dropout rates are low compared to other TFTs (Jericho et al., 2022; Raghuraman et al., 2021). NET is known to reach its largest effects in particular in the months following the last session (Elbert et al., 2015; Siehl et al., 2021).

Based on the findings that NET is particularly suitable for patients with multiple traumas and can be effective even under suboptimal psychosocial conditions, we consider NET a promising approach for developing an intensive treatment programme in our clinic, a tertiary centre for the treatment of complex post-traumatic psychopathology. NET distinguishes itself from other therapies by focusing on the entire lifespan and establishing connections between events. We assume that these connections are more easily formed in an intensive format. Another important difference between NET and other forms of trauma-focused treatment is that positive experiences are explicitly involved in the construction, discussion, and testimony report of the lifeline. This may give a more balanced perspective on life and may increase adherence, also when being applied in an intensive format. The aim of this pilot study was therefore to investigate a highly intensive 10-day treatment programme with NET as primary therapeutic method in an inpatient department. We explored changes in clinician-rated and self-reported PTSD symptoms, symptoms of depression and general psychopathology after completing this programme with two NET sessions and one trauma-sensitive yoga session per day as well as other supportive elements. Based on the literature on highly intensive trauma treatment and on regular NET treatment, we expected the HI-NET programme to be feasible and to result in substantial effects on PTSD and related symptomatology especially at the endpoint 4 months post-treatment.

## 2 Materials and methods

### 2.1 Participants

Participants were patients who were referred for treatment at ARQ Centrum’45, a tertiary centre for diagnosis and treatment of complex psychotrauma-related disorders. Eligibility criteria for the study were: (1) being at least 18 years old; (2) having a diagnosis of PTSD as assessed with the Clinician Administered PTSD scale for DSM-5 (CAPS-5; Boeschoten et al., 2018; Weathers et al., 2018); (3) being motivated to undergo a brief and highly intensive inpatient trauma treatment; (4) able to communicate in Dutch, English or their native language if there was an interpreter available; and (5) having a crisis plan in place and engaged in ongoing treatment, which they could continue after completion of the 10-day programme. Exclusion criteria were: (1) acute suicidality, (2) severe or acute psychotic state, (3) severe alcohol or substance dependence, or (4) severe somatic complaints consistent with an organic brain disorder or a somatic condition that constitute a contraindication for exposure treatment according to a physician (e.g., a heart condition for which additional stress should be avoided).

### 2.2 Procedure

After referral, an intake for HI-NET took place. During intake, we considered the patient’s cultural background and used elements of the Cultural Formulation Interview (Rohlof et al., 2017) where appropriate. In case of a language barrier, we made use of a certified interpreter. Our diagnostic process took into account the specific context of the individual, whether refugee, police officer, or veteran. Our staff has routinely received continuing education and intervision in context-specific knowledge about the patient groups who seek treatment at our centre. During the intake, patients were provided with both verbal and written information regarding the treatment, measurements, and the study. The eligibility and exclusion criteria were also reviewed. Patients gave their written informed consent to participate in the study and for the use of their data. All participants underwent a medical check-up by the ward physician. Before, during and after treatment, the referring therapist was contacted to ensure optimal embedding in ongoing treatment.

A baseline measurement (T0) took place on average 5.47 days before treatment (SD = 3.13), a post-treatment assessment (T1) on average 11.05 days after treatment (SD = 11.63), and a follow-up assessment (T2) on average 113.32 days after treatment (SD = 29.01), which is approximately 3.8 months. Since this study only included measurements that are also used in regular care, review by the medical ethics committee was not deemed necessary.

### 2.3 Treatment

Patients were admitted to the inpatient department of ARQ Centrum’45 on Sunday evening and discharge was 2 weeks later on Saturday. Treatment was from Monday to Friday. During the weekend, patients stayed in the clinic without therapy. The programme consisted of two daily TFT sessions, with a total of 16 NET sessions according to the protocol (Jongedijk, 2021; Schauer et al., 2011) and four NET-based trauma-focused art therapy (AT) sessions. One trauma-sensitive yoga (TSY) session was offered per day, as well as 15 min of personal support from a nurse. Throughout the programme the nurses also stimulated conversation and shared social activities. After each NET session, patients were encouraged to go for a walk or exercise. If patients did not sufficiently understand Dutch or English, an interpreter was arranged, preferably one who was familiar with NET. During the lifeline session, the interpreter was physically present. During the subsequent exposure sessions, a telephone interpreter translated. The number of sessions and the duration of the programme were based on the NET protocol which recommend 8–12 sessions and more sessions if needed for treatment of multiple traumatic events. If more than 14 events were placed on the lifeline, patients were asked to make a selection of the most debilitating or important events which is a procedure according to the NET treatment protocol (Jongedijk, 2021; Schauer et al., 2011).

NET treatment was delivered according to the Dutch protocol (Jongedijk, 2021) by two trained NET therapists who were professionals in mental health care, who had been trained according to the Dutch requirements to deliver NET treatment and rotated during the programme. A total of nine therapists delivered the treatment. The sessions were attended by a nurse with clinical experience in trauma treatment, who did not participate in the therapy but only was present in the therapy room to write the testimony.

During the first session, the lifeline was laid out with stones for traumatic events, flowers for positive and resilient events, candles for grief situations and sticks for aggressive acts. In the following manualized sessions, the lifeline was processed in chronological order, starting from birth, by applying NET exposure techniques to the traumatic events and recounting the resilient events. During these NET sessions, the therapist focused on the images, sounds, smells, physical reactions and emotions associated with the traumatic event. In this way, the “cold” (explicit or factual) memories were integrated with the “warm” (implicit) memories to give the traumatic memories context in time and space and to embed them within autobiographical memory (Elbert et al., 2015). During the final NET session, the full testimony was read out loud and handed to the patient. If possible, a loved one was present at this session. This was preceded by a discussion by therapist and patient if this would be helpful and if so, if it would be appropriate to share with the loved one, depending on his or her vulnerability.

Trauma-sensitive yoga (TSY) was added to the programme to reduce the tension evoked by the trauma-focused sessions and promote relaxation between sessions, as well as emotion regulation and body awareness (Kananian et al., 2017; Yehuda et al., 2017). The TSY protocol was based on ‘Trauma-sensitive yoga’ (Emerson and Hopper, 2011) and the ‘Yoga for the mind’ programme (Mason, 2011), consisting of postures, breathing exercises and guided meditation. The sessions were kept safe, easy and predictable through low stimuli and relatively slow movements. No music was used. The instructor’s language was inviting, encouraged curiosity about bodily sensations and made people aware of stress-induced bodily sensations.

Art therapy (AT) was integrated into the programme as a complementary therapeutic approach, allowing patients to engage with and benefit from Narrative Exposure Therapy (NET) through creative expression. During art therapy (AT) sessions, patients engaged in narrative exposure focused on an event from their lifeline, such as a flower or a stone, or elaborated on an event that had already been processed in a Narrative Exposure Therapy (NET) session. While the primary focus was on visual elements such as drawing, painting, or sculpting to support the narrative, patients also told about their work in the sessions. Clinical experience has demonstrated that patients who struggle to articulate their experiences verbally during narrative exposure are often able to more fully access, express, and process their traumatic memories through the creative and expressive processes facilitated by AT. This integration underscores the added value of AT in enhancing therapeutic outcomes for individuals with trauma-related issues.

As an illustration of the therapy, we provide a case description of one patient.

### 2.4 Statistical analyses

Firstly, standard quantitative analyses were applied for all instruments to investigate differences between T0 and T1 and T0 and T2 at group level. We used paired t-tests to analyse symptom reduction over time, given the small sample size (*N* = 19 at follow-up) and the exploratory nature of this pilot study. Other types of analysis such as mixed models require larger datasets for stable estimates, and assumptions of repeated measures ANOVA may not hold in small samples. Preliminary effect sizes (Cohen’s *d*) were calculated using means and standard deviations of total scores of the questionnaires from T0–T1 and T0–T2. Cohen’s d was computed using the standard formula:

d = M2 − M1SDpooledd = \frac{M_2 - M_1}{SD_{\text{pooled}}}d = SDpooledM2 − M1 where M1M_1M1 and M2M_2M2 are the group means at two time points, and SDpooledSD_{\text{pooled}}SDpooled represents the pooled standard deviation.

To complement group-level comparisons, we also calculated Reliable Change Index (RCI; Jacobson and Truax, 1991) values to assess individual treatment effects. RCI was applied for all instruments to investigate whether there was a statistically reliable change on an individual level between the abovementioned measurement points. RCI was calculated using the Jacobson and Truax (1991) formula, which determines whether an individual’s symptom change exceeds measurement error:

RCI = X2 − X1SERCI = \frac{X_2 – X_1}{S_E}RCI=SEX2 − X1 where X1X_1X1 and X2X_2X2 represent an individual’s pre-and post-treatment scores, and SES_ESE (standard error of measurement) was derived from test–retest reliability values.

If RCI values were > + 1.96 or < −1.96 it can be concluded that the difference between the test scores is statistically reliable, i.e., there is a 95% certainty that the difference between test scores is due to actual change, and not due to measurement error. RCI values were calculated with Cronbach’s alpha. Due to the small sample size Cronbach’s alpha was not calculated but taken from existing literature: 0.90 for the CAPS-5 (Boeschoten et al., 2018), 0.95 for the PCL-5 (Bovin et al., 2016), 0.92 for the BDI-II (Beck et al., 2002) and 0.84 for the BSI (Bormann et al., 2013). Based on the RCI values, patients were categorised in one of three categories of treatment outcomes from T0-T1 and from T0-T2: (1) improved (a RCI value smaller than −1.96); (2) unchanged (a RCI value between −1.96 and 1.96); and (3) worsened (a RCI value larger than 1.96). We also explored treatment outcome based on RCI for the subgroups of refugees and patients of Dutch origin.

### 2.5 Instruments

#### 2.5.1 Clinical interviews

The Dutch version of the Clinician Administered PTSD scale for DSM-5 (CAPS-5) was used to assess the PTSD diagnosis and severity of PTSD symptoms over the past month (Boeschoten et al., 2018; Weathers et al., 2018). The CAPS-5 has shown strong interrater reliability and test–retest reliability on both the PTSD diagnosis and the total severity score. The Cronbach’s ɑ of the Dutch CAPS-5 is 0.90 (Boeschoten et al., 2018) and 0.88 for the original version (Weathers et al., 2018), which is considered excellent.

#### 2.5.2 Self-report questionnaires

##### 2.5.2.1 PCL-5

The PTSD Checklist for DSM-5 (PCL-5) is a self-report measure that assesses DSM-5 symptoms of PTSD over the past week. The purpose of administering the PCL-5 was monitoring change before, during and after treatment. The psychometric evaluation of the Dutch translation demonstrated high internal consistency (Cronbach’s alpha = 0.95) and good validity (Boeschoten et al., 2015).

##### 2.5.2.2 BSI

The Brief Symptom Inventory (BSI) is a self-report questionnaire designed to assess the nature and severity of general psychopathology. The BSI consists of 53 items covering nine symptom dimensions: somatic complaints, cognitive problems, interpersonal sensitivity, depressive symptoms, anxiety, hostility, phobic fear, paranoid ideation and psychoticism (Derogatis and Melisaratos, 1983). It is a well validated instrument that rates the extent of being bothered by various mental health symptoms in the past week. It has good psychometric qualities: the internal consistency is good (Cronbach’s alpha = 0.84), and validity is sufficient (Bormann et al., 2013). The Dutch version of the BSI has an acceptable reliability and validity as well (De Beurs and Zitman, 2006).

##### 2.5.2.3 BDI-II

The Beck Depression Inventory-II (BDI-II-NL) (Beck et al., 2002; van der Does, 2002) is a self-report questionnaire for measuring the severity of depressive symptoms. It is composed of items relating to symptoms of depression as well as physical symptoms related to depression. The Cronbach’s ɑ is 0.92 for the Dutch BDI-II (Beck et al., 2002).

## 3 Results

A total of 28 participants enrolled in the current pilot study. Baseline demographic and clinical characteristics for this full sample are shown in Table 1. Five patients discontinued the treatment prematurely for varying reasons (see paragraph 3.1). Additionally, six participants were excluded from the final analysis because they either declined to complete post-treatment assessments after treatment dropout (*n* = 5, 17.9%) or could not be contacted (*n* = 1; 3.6%). Regarding dropout in the subgroups we explored, 1 out of 9 refugees (11%) and 7 out of 19 patients from Dutch origin (37%) dropped out in the course of the study.

**Table 1:** Baseline demographic and clinical characteristics of treatment sample (*N* = 28).

Characteristic	Category	*N* (%)	M (*SD*)
Sex	Male	18 (64)	
	Female	10 (36)	
Civil status	Single	8 (29)	
	Married/partnership	7 (25)	
	Married/partnership with children	8 (29)	
	Single with children	5 (18)	
Educational level^a^	Low	8 (29)	
	Middle	9 (32)	
	High	4 (14)	
	Unknown	7 (25)	
Trauma background	Childhood trauma	9 (32)	
	Refugee/asylum seeker	9 (32)	
	Profession-related		
	Veteran	5 (18)	
	Police	4 (14)	
	Other	1 (4)	
Duration of PTSD, mean (SD), years			14.93 (16.32)
Age, years			45.79 (12.51)
Previous treatment		28 (100)	
	Pharmacotherapy	21 (75)	
	Psychotherapy	20 (71)	
	Trauma-focused therapy		
	EMDR	11 (39)	
	BEPP	1 (0)	
	Imaginal exposure	1 (0)	
	Other	2 (1)	
	Group therapy	8 (29)	
Co-morbid disorders based on intake data		21 (75)	
	Depressive disorder	17 (60)	
	Substance abuse	7 (25)	
	Personality disorder	4 (14)	
	Other	7 (25)	
Interpreter required during HI-NET		4 (14)	

a Low: completed elementary school or lower vocational education.

Middle: completed high school or middle level vocational education.

High: completed high level vocational education, pre-university, college or university degree.

For the subset of 19 participants who were included in the final analysis, mean scores for CAPS-5, PCL-5, BDI-II, and BSI across all time points are reported in Table 2, along with categories of treatment outcome at follow-up (improved, unchanged, and worsened). The sample sizes for each scale at each time point are also detailed in the description of Table 2.

**Table 2:** Mean scores of total sample (*N* = 19) and in categories of treatment outcome at follow-up.

Instrument		T0 Mean (SD)	T1 Mean (SD)	T2 Mean (SD)
CAPS-5 total		46.23 (8.98)	44 (9.65)	37.68 (13.22) ^b^
	Improved (*N* = 8)	48.38 (10.72)	42.88 (10.44)	28.50 (14.26)
	Unchanged (*N* = 10)	45.90 (9.12)	44.10 (10.77)	43.30 (6.91)
	Worsened (*N* = 1)	41.00 (0)	50.00 (0)	55.00 (0)
PCL-5 total		53.77 (8.36)	50.14 (9.97)	44.00 (14.77) ^c^
	Improved (*N* = 11)	56.10 (5.76)	50.20 (8.48)	36.50 (15.49)
	Unchanged (*N* = 7)	53.71 (6.60)	52.57 (13.59)	53.14 (6.84)
	Worsened (*N* = 1)	49.00 (0)	50.00 (0)	55.00 (0)
BSI total		2.07 (0.62)	2.49 (2.66)	1.66 (0.72) ^b^
	Improved (*N* = 5)	2.36 (0.68)	2.05 (1.13)	0.94 (0.56)
	Unchanged (*N* = 13)	1.98 (0.64)	1.97 (0.56)	1.91 (0.61)
	Worsened (*N* = 1)	1.25 (0)	1.04 (0)	2.00 (0)
BDI-II total		35.86 (8.35)	31.05 (11.32) ^a^	28.11 (12.35) ^b^
	Improved (*N* = 6)	38.17 (9.33)	30.00 (16.64)	17.67 (13.14)
	Unchanged (*N* = 13)	33.33 (10.83)	29.00 (9.97)	30.83 (8.61)

CAPS-5, Clinician-Administered PTSD Scale; PCL-5, PTSD Check List for DSM-5; BSI, Brief Symptom Inventory; BDI-II, Beck Depression Inventory-II. T0, baseline; T1, post-treatment; T2, four-month follow-up. Improved, RC ≤−1.96; unchanged, RC>−1.96 or <+1.96; worsened, RC> + 1.96. Unless otherwise specified, *N* = 19; ^a^ *N* = 17; ^b^ *N* = 16; ^c^*N* = 15.

### 3.1 Feasibility

As mentioned above, five patients dropped out from treatment (17.9%). In two patients, inappropriate behaviour occurred, with one patient engaging in sexually inappropriate behaviour towards another patient, and the other patient engaging in rudeness and offensive language towards the staff. These were grounds for these patients to be further excluded from the treatment programme. During the pilot there was the outbreak of the SARS-CoV-2 infection this resulted in two patients having to leave the clinic due to the Dutch covid policy. The final patient indicated that he had lost interest to continue the programme due to changed priorities.

For the remainder of the participants there were no missed sessions. The tolerability of the sessions was good. Standard therapists’ assessments which were part of clinical care indicated that dissociative symptoms and suicidality did not increase during the treatment in any of the patients. No crisis assessments by a psychiatrist were required either. During the post-assessments patients generally expressed themselves positively about the treatment, stated that they felt acknowledged through the application of NET and that the design of the programme helped them to go deeper into the narrative exposure to the traumatic memories than in previous treatments. One patient showed deterioration in PTSD on the PCL-5 and another patient on the CAPS-5 from baseline to post-treatment, probably due to highly stressful psychosocial circumstances and stressors in private life which were present for both patients.

### 3.2 PTSD symptoms

A statistically significant decrease in CAPS-5 scores was found from baseline (*M* = 46.68, SD = 9.47) to four-month follow-up (*M* = 37.68, SD = 13.22), *t* (19) = 3.51, *p* = 0.003, with a large effect size (Cohen’s *d* = 0.80). In addition, a statistically significant decrease in PCL-5 scores was found from baseline (*M* = 55.12, SD = 6.04) to four-month follow-up (*M* = 43.35, SD = 14.96), *t* (16) = 3.47, *p* = 0.003, with a large effect size (Cohen’s *d* = 0.84). From baseline to post-treatment there was no statistically significant decrease on the CAPS-5 nor on the PCL-5.

Table 3 shows the reliable change index per patient. For the CAPS-5, six patients showed an improvement consistent with a reliable change from T0 to T1, and eight patients showed an improvement consistent with reliable change from T0 to T2. Of the eight patients who showed a reliable change at follow-up, three patients had lost their PTSD diagnosis at follow-up. Ten patients remained unchanged at follow-up compared to baseline. Notably, one patient worsened from baseline to follow-up on the CAPS-5.

**Table 3:** Treatment outcome and reliable change indexes (RCI) on Clinician-Administered PTSD Scale (CAPS-5) scores per patient from T0 to T1 and from T0 to T2.

Patient number	CAPS-5 total T0-T1	RCI T0-T1	CAPS-5 total T0-T2	RCI T0-T2
4	2	0.94	1	−5.43
9	1	−2.60	1	−4.72
22	2	1.42	1	−7.56
6	1	−2.13	1	−4.72
8	1	−3.78	1	−3.07
10	2	0.24	1	−3.31
12	2	−1.42	1	−4.01
15	1	−3.07	1	−4.72
1	2	0	2	0.94
2	2	0	2	−0.94
3	1	−4.72	2	−1.89
5	2	−0.47	2	−1.18
13	3	3.31	2	0
14	1	−3.07	2	0
17	2	−0.44	2	−0.94
19	2	−1.42	2	−0.47
20	2	0.94	2	−1.65
21	2	1.65	2	0
18	3	2.13	3	3.31
7	2	−0.75		
11	3	2.49		
16	2	−0.75		

T0, baseline; T1, post-treatment; T2, 4-month follow-up. Improved, RC ≤ −1.96; unchanged, RC >−1.96 or <+1.96; worsened, RC> +1.96. 1, improved (RC ≤ −1.96); 2, unchanged (RC > − 1.96 or < +1.96; 3, worsened (RC > + 1.96). Cronbach’s alpha = 0.90 (Boeschoten et al., 2018).

For the PCL-5, nine patients showed an improvement consistent with a reliable change from T0 to T1, and ten patients showed improvement consistent with reliable change from T0 to T2. Of the ten patients who showed a reliable change at follow-up, three patients had lost their probable PTSD diagnosis at follow-up. Seven patients remained unchanged in their PCL-5 scores at follow-up compared to baseline. One patient worsened from baseline to follow-up on the PCL-5 (a different patient than the one that showed worsening on the CAPS-5).

To explore potential differences in treatment response between refugees and patients from Dutch origin, RCIs of these two subgroups on CAPS-5 and PCL-5 were reported as well. Of the subset of 19 participants included in the final analysis, 4 out of 7 refugees (57%) showed improvement consistent with reliable change on CAPS-5 from T0 to T2. Of the patients from Dutch origin, 4 out of 12 (33%) showed improvement consistent with reliable change on CAPS-5 from T0 to T2. On the PCL-5, 5 out of 7 refugees (74%) demonstrated improvement consistent with reliable change from T0 to T2, whereas this proved to be the case for 5 out of 12 patients from Dutch origin (42%).

To provide further insight into changes in PTSD subdomains, descriptive statistics for the subscales of the CAPS-5 and PCL-5 were calculated and reported. For the CAPS-5 subscales, the mean scores and standard deviations for re-experiencing symptoms (B criterion) were 11.71 (SD = 3.12) at T1, 12.13 (SD = 2.64) at T2, and 9.56 (SD = 1.59) at T3. For avoidance symptoms (C criterion), the mean scores were 4.79 (SD = 1.93) at T1, 5.87 (SD = 1.77) at T2, and 5.44 (SD = 1.67) at T3. For negative alterations in cognitions and mood symptoms (D criterion), the mean scores were 14.64 (SD = 5.55) at T1, 14.73 (SD = 4.53) at T2, and 11.00 (SD = 5.59) at T3. For alterations in arousal and reactivity symptoms (E criterion), the mean scores were 11.71 (SD = 2.67) at T1, 10.93 (SD = 3.30) at T2, and 10.00 (SD = 2.06) at T3. For the PCL-5 subscales, the mean scores and standard deviations for re-experiencing symptoms (B criterion) were 13.78 (SD = 2.84) at T1, 14.18 (SD = 2.96) at T2, and 10.17 (SD = 4.79) at T3. For avoidance symptoms (C criterion), the mean scores were 5.72 (SD = 1.84) at T1, 4.94 (SD = 1.82) at T2, and 5.17 (SD = 2.48) at T3. For negative alterations in cognitions and mood symptoms (D criterion), the mean scores were 18.06 (SD = 4.87) at T1, 17.71 (SD = 4.51) at T2, and 15.33 (SD = 3.72) at T3. For hyperarousal (E criterion), the mean scores were 15.22 (SD = 3.44) at T1, 14.12 (SD = 3.26) at T2, and 13.67 (SD = 4.84) at T3.

### 3.3 Depressive symptoms

A statistically significant decrease in BDI-II scores was found from baseline (*M* = 35.72, SD = 8.58) to post-treatment (*M* = 30.17, SD = 11.57), *t* (17) = 2.35, *p* = 0.031, with a medium effect size (Cohen’s *d* = 0.55), and from baseline (*M* = 35.56, SD = 8.571) to four-month follow-up (*M* = 27.39, SD = 12.30), *t* (17) = 3.29, *p* = 0.004, with a large effect size (Cohen’s *d* = 0.78).

In terms of BDI-II scores, seven patients reliably improved from baseline to post-treatment, and six patients reliably improved at follow-up. Four out of these six patients scored below the clinical cutoff at follow-up. Thirteen patients showed no change at follow-up compared to baseline. Three patients showed worsening at post-treatment, but no patients showed worsening at follow-up compared to baseline.

### 3.4 General psychopathology

No significant change in BSI scores was found from baseline to post-treatment, nor from baseline to four-month follow-up. Regarding BSI scores, four patients showed improvement from T0 to T1, and five patients showed improvement consistent with a reliable change from T0 to T2. Compared to baseline, thirteen patients showed no change at follow-up and one patient showed worsening at follow-up compared to baseline. Inspection of the subscales of the BSI showed that improvements were most prominent on the subscales depression, cognitive difficulties, phobic anxiety, fear, and hostility whereas scores on the other subscales remained unchanged.

## 4 Discussion

This pilot study investigated the feasibility and preliminary effectiveness of a ten-day highly intensive inpatient treatment programme for PTSD with NET as primary trauma-focused treatment component and additional components of AT and TSY in patients who had all received previous treatment for their disorder. For patients who completed the programme we found significant, large-sized improvements in clinician-rated and self-reported PTSD symptom severity and depressive symptom severity at four-month follow-up but not for general psychopathology. Changes in clinician-rated and self-reported PTSD symptom severity followed comparable patterns: there was no improvement immediately post-treatment but there were significant improvements at follow-up 4 months post-treatment. Depressive symptomatology already improved significantly at post-treatment, and this improvement strengthened at 4-month follow-up. These findings are in line with a recent case series on a one-week intensive NET format, which found similar patterns of PTSD and depressive symptom decrease up to 6 months post-treatment (Miller et al., 2024). In terms of reliable changes, 53% of the patients who completed the programme demonstrated improvement in self-reported PTSD and one patient worsened on self-reported PTSD. NET in an intensive form, and embedded in an inpatient programme, appears to be feasible and the dropout, which was mostly due to external circumstances, seems comparable to that of regular trauma-focused treatment. In the post-assessments and follow-up assessments, patients reported the tolerability of the programme to be good.

Although group-level changes were large-sized, we note that these are representative of treatment completers because we were not able to follow up the patients who prematurely dropped out. On an individual level, slightly more patients fulfilled criteria for improvement consistent with reliable changes in self-reported PTSD than in clinician-rated PTSD. This may be explained by generally higher baseline scores on the PCL-5 as compared to CAPS-5 as documented in literature (Lee et al., 2024), leaving more room for improvement over the course of treatment and in the following months. We also observed that the decreases in PTSD symptom severity during the 10-day programme were somewhat smaller than the effects of a standard weekly NET (Lely et al., 2019; Siehl et al., 2021), whereas the decrease in depressive symptoms in our study compares favourably with that of weekly NET in recent meta-analyses (Lely et al., 2019; Siehl et al., 2021). The preliminary treatment effects in this study are comparable to pilot results of other high-intensity treatment forms with TFT such as EMDR and TF-CBT (Hendriks et al., 2018; Zepeda Méndez et al., 2018) but smaller than those found in large-scale randomised studies of high-intensity treatment programs (Ehlers et al., 2014; Foa et al., 2018; Oprel et al., 2021; Dell et al., 2023; Voorendonk et al., 2023). A factor likely playing a role in the relatively modest effect sizes is that patients in our sample stem from a highly specialised, tertiary centre for the treatment of trauma-related psychopathology. As such, the patients in our pilot all had a history of several previous unsuccessful treatment indicating a degree of treatment resistance (Nijdam et al., 2023). As the average duration of PTSD symptoms in our sample was almost 15 years, factors related to chronicity of symptoms may also have played a role in the attenuated effects including a high prevalence of several comorbid diagnoses (Jongedijk et al., 2019, 2020; Nijdam et al., 2023), as detailed in the description of the sample.

Looking at the individual level, patients who did not improve according to their RCI largely remained stable regarding their symptomatology, except one patient who showed deterioration in self-reported and another in clinician-rated PTSD symptoms. Suddenly emerging lawsuits and unexpected gaps in follow-up treatment may have played a role here. We also observed considerable variety in individual RCIs in response to the programme, warranting further research to determine for whom this programme may be most helpful. In the future, this could lead to more refined criteria for admission, enhancements to the programme, and a deeper understanding of which aspects are most effective. An important finding of our pilot study concerns the feasibility of a highly intensive programme in which NET was offered twice a day. During post-and follow-up assessments, patients indicated satisfaction with the treatment programme, felt acknowledged in their experiences and expressed that the programme facilitated them to share more details of their traumatic experiences than in previous treatments. No increases in suicidality or dissociative symptoms were observed during the pilot.

We believe our pilot study adds to the evidence base by describing and exploring effects of a novel form of intensified treatment. Based on the findings, we think NET can successfully be converted to a short highly intensive programme, in our case performed in an inpatient setting with additional therapeutic elements. Earlier research on NET has largely been conducted in outpatient settings without additional therapies. However, one previous study has already shown the feasibility and preliminary effectiveness of integrating NET with elements of art therapy and body-oriented interventions in a day treatment setting for refugees (De la Rie et al., 2020). Another pilot study for highly burdened patients with borderline personality disorder and PTSD found the combination of NET with supportive one-to-one sessions, art or music therapy, body therapy, and movement therapy to be feasible in an inpatient setting (Steuwe et al., 2016). Several highly intensive therapy programs also integrate other trauma-focused treatments with trauma-sensitive yoga, exercise or art therapy with good results (Zepeda Méndez et al., 2024; Voorendonk et al., 2023; Auren et al., 2022). What distinguishes NET from more commonly used trauma treatments is that this approach has a different theoretical framework: NET does not focus solely on intrusive memories but in particular on the elaboration of autobiographical memory systems. Ultimately, this will lead to an integrated perspective on the patient’s life history as a whole, with all good and the bad events experienced and especially the connections between them. These unique characteristics of NET may also explain why the intervention exerts its strongest effects several months after treatment. The alteration of the fear network and the resulting reprocessing of similar traumatic events may continue in the aftermath of therapy (Elbert and Schauer, 2002; Siehl et al., 2021). As NET has specifically been designed for patients with multiple traumas, the programme may be especially suitable for refugees, war and torture survivors, as well as patients who have experienced repeated and/or prolonged occupational trauma exposure or for patients with adverse childhood experiences. While our findings suggest that intensive NET is feasible and yields comparable outcomes to standard NET, the necessity of this intensive format requires further investigation. Future research should investigate whether intensive NET provides distinct advantages, such as faster symptom reduction, improved cost-effectiveness, or greater accessibility for patients who may struggle with once-weekly treatment schedules. Further research is also needed to determine whether this approach has added value for specific patient groups as compared to other intensified trauma-focused psychotherapies. Explorative findings of our study showed a tendency for a greater proportion of refugees to respond to the program than of patients with Dutch origin, which is in line with research indicating the suitability of NET specifically for refugees (Siehl et al., 2021; Raghuraman et al., 2021). For the applicability across culturally diverse populations, we deem it important to understand how language barriers and cultural differences potentially impact treatment outcomes in a highly intensive format with NET.

### 4.1 Limitations

The most important limitation of our study concerns the lack of a control group. Therefore, we cannot rule out possible time effects and effects of nonspecific therapeutic factors of the programme. The programme is a combined intervention consisting of NET, AT, TSY, and supportive interventions in the context of an inpatient setting. Because we explored the effects of the programme as a whole, we cannot draw conclusions regarding the relative contribution of the different elements to the effects. Dismantling studies may shed light on the contribution of separate trauma-focused and non trauma-focused components. We also acknowledge the small sample size and the missing assessments, especially of patients who dropped out. Although we assessed both clinician-rated and self-reported PTSD post-treatment at 4 months after treatment, further long-term follow-up assessments at 6 and 12 months post-treatment could have given a better indication of the progression in changes over time, as some meta-analyses indicate that the effects of NET on PTSD become more pronounced over that time period (Raghuraman et al., 2021; Siehl et al., 2021).

## 5 Conclusion

The primary aim of this study was to investigate a newly developed 10-day highly intensive treatment programme for patients with PTSD, consisting of NET, AT and TSY, in terms of feasibility and preliminary effects. The large effects on clinician-rated and self-reported PTSD symptom severity as well as depressive symptom severity we found at four-month follow up are promising, as all patients had a history of previous treatment for their PTSD indicating a degree of resistance to standard interventions. Dropout was comparable to weekly trauma-focused treatment and the tolerability of the programme was good according to the patients and therapists who participated. We therefore conclude that NET can be adapted into a short highly intensive treatment programme within an inpatient setting. Future studies are encouraged to investigate the effects of highly intensive forms of NET further, preferably in comparison to standard care conditions and with longer follow-up periods in multiple baseline or randomised controlled designs. These studies could also investigate whether highly intensive NET is especially beneficial for patients suffering from PTSD resulting from repeated and/or prolonged forms of trauma. Furthermore, narrative assessment tools and qualitative methods may be helpful to elucidate changes in patients’ autobiographical narratives and may provide important insights into the therapeutic processes in a highly intensive NET programme. An important clinical impression we had during this project was that aftercare and active contact with the referring clinician was important in sustaining the effects of the programme, especially in case of multiple psychosocial stressors. Our programme as well as other highly intensive programs may contribute to the needs of patients by accelerating change. Hopefully our pilot study can serve to encourage future work in this area.

## Data Availability

The data that support the findings of this study are available from the corresponding author, PS, upon reasonable request. Data were collected primarily for clinical purposes and are not deposited in a community recognized repository because participants have not provided informed consent for sharing data outside of the institute.

## Appendix: Case study

James, a 35-year-old man, grew up in a rural area near a major South American city, where he faced significant family turmoil and adversity from a young age. After witnessing his parents’ divorce, he was sent to live with his paternal grandparents and had to work apart from his mother under challenging conditions. When his grandparents were no longer able to care for James, he started a search to find his mother. Because of his mother’s poor financial circumstances and poor health, they were forced to live in one of the slums. In this slum he was a victim of violence but also witnessed it as well as very serious criminal activities. His talent for sports made it possible for him to get education and eventually leave his homeland for Europe.

When James immigrated to Europe, his challenges persisted. He suffered a severe and traumatic workplace injury and struggled to adapt to his new environment. The combination of his traumatic past and these new hardships triggered severe symptoms of PTSD and depression. Determined to find effective treatment for his trauma and depression, James was introduced to the intensive Narrative Exposure Therapy (NET) in a clinical setting. The intensive nature of the therapy left no room for avoidance; James had to confront his past head-on. He meticulously created his lifeline in a single session and selected the most significant moments of his life. His lifeline consisted of 22 events: one event symbolising loss, depicted by a candle; seven beautiful moments, represented by flowers; and 13 traumatic events, illustrated as stones.

James underwent the highly intensive programme which involved 16 sessions over the course of 2 weeks. This approach allowed for an elaborate, ongoing examination and processing of his memories. Unlike previous treatments for his PTSD and depression which were unsuccessful due to psychosocial stress in between sessions and reduced match with the rationale, the sessions were so frequent that James was able to see clear links between his traumatic experiences and his reactions to these which increased his motivation. The frequency of the sessions combined with his stay in the clinic, left him with minimal daily distractions and he was able to fully immerse himself in the therapeutic process.

Through the programme, James was able to place his early and adult traumatic experiences in context, helping him understand how these events shaped his responses to anxiety-provoking situations throughout his life. This newfound understanding enabled James to manage and reduce his anxiety and depression more effectively, resulting in a significant improvement in his overall well-being. For the first time, James felt truly heard, especially since the sessions were facilitated by an interpreter due to language barriers. He reported that the intensity of the treatment and the structured environment of the clinic contributed to the valuable breakthroughs he experienced. James experienced significant improvements in his PTSD symptoms, ultimately losing his PTSD diagnosis and experiencing substantial relief from depressive symptoms.
